# Developing a questionnaire to explore lay people’s preferences for communicating hereditary conditions within families: insights from a cognitive interview study

**DOI:** 10.1007/s12687-025-00783-6

**Published:** 2025-03-18

**Authors:** Lea Godino, Linda Battistuzzi, Liliana Varesco, Daniela Turchetti, Vanessa Gentili, Paolo Chiari, Alvisa Palese

**Affiliations:** 1 Medical Genetics Unit, IRCCS Azienda Ospedaliero-Universitaria Di Bologna, Bologna, Italy; 2 Department of Biomedicine and Prevention, University of Rome “Tor Vergata”, Rome, Italy; 3 Medical Oncology Unit 2, IRCCS Ospedale Policlinico San Martino, Genoa, Italy; 4 Unit of Hereditary Cancer, IRCCS Ospedale Policlinico San Martino, Genoa, Italy; 5 Department of Medical and Surgical Sciences, University of Bologna, Bologna, Italy; 6 Department of Medicine, University of Udine, Udine, Italy

**Keywords:** Cognitive interview, Public, Genetic testing, Genetic literacy

## Abstract

Cognitive interviews are a valuable qualitative method for developing and refining survey instruments, particularly on complex topics such as genetic health. They help address misunderstandings between intended meanings and respondent interpretations, enhancing data validity and ensuring comprehensibility. This study aimed to refine a questionnaire exploring the attitudes and preferences of the Italian general population regarding the communication of potential hereditary conditions within families. Through iterative testing, issues related to questionnaire instructions, question wording, and the sensitive nature of the topics were identified and addressed. Most concerns emerged in the first round of cognitive interviews, while the second round only required minor refinements. The qualitative analysis identified four key themes reflecting participants’ challenges in understanding genetic information: (1) difficulties with genetic terminology, including gene names and scientific jargon, which induced anxiety and hindered comprehension; (2) ambiguities surrounding the terms "genetic testing" and "family," with confusion about the nature of genetic testing and the scope of "family" in genetic contexts; (3) misinterpretations of "genetic risk" as an existing disease diagnosis rather than a probabilistic concept, leading to misunderstandings about the implications of genetic predisposition; and (4) conflation of "authorization" and "responsibility" in genetic communication, further complicated by uncertainty regarding privacy and confidentiality. Findings from this study informed targeted modifications to the questionnaire to enhance its clarity and accessibility. Our study highlights the importance of cognitive interviewing in refining survey tools on genetic communication, ensuring that such instruments effectively capture public perceptions and facilitate informed decision-making**.**

**Supplementary Information:**

The online version contains supplementary material available at 10.1007/s12687-025-00783-6.

## Introduction

A genetic predisposition is an increased likelihood of developing a particular disease that is associated with specific genetic variants, which are often inherited from a parent. For individuals with an inherited predisposition, having genetic testing and learning that they carry a pathogenic variant (PV) can have great clinical and personal utility, e.g. in terms of targeted treatment (Cortesi et al. 2021), preventive measures (Sessa et al. 2023; Nherera et al. 2011), informed reproductive decisions and life planning (Godino et al. 2016; Bertonazzi et al. 2022). It can also have important consequences for their bloodline relatives, who are also at risk of carrying the PV and thus of developing the disease. For this reason, healthcare professionals generally encourage patients who carry a PV to discuss the implications of their test result with their relatives, so that they can also seek genetic testing, if they so choose (Frey et al. 2022). The systematic process whereby genetic testing identifies individuals within a family who have inherited a genetic risk is termed cascade testing.

The uptake of cascade testing is reported to be suboptimal even for conditions in which its clinical utility has been shown to be high (Frey et al. 2022; Trevisan et al. 2024) The process of cascade testing involves multiple communication steps, from healthcare providers to patients and from patients to their relatives. Although low cascade testing rates should not be exclusively attributed to communication difficulties, as some relatives may be informed but choose not to pursue testing nonetheless, how information is conveyed, received, and interpreted at each step can promote or interfere with relatives’ access to reliable and accurate information (Pedrazzani et al. 2022).

Within families, studies have shown that feelings of guilt, anxiety or blame, concerns about privacy and discrimination, and a lack of sense of responsibility (Afaya et al. 2024; Levine et al. 2024), and genetic fatalism (Ponz de Leon et al. 2004), can all impact patients’ decision to disclose genetic risk information.

Another factor that is likely to play a prominent role in genetic risk communication within cascade genetic testing is genetic literacy, defined as an individual’s ability to understand and use genetic information (Boerwinkel et al. 2017), given that limited familiarity with genetic concepts can interfere with grasping notions of genetic risk and the purpose, utility and implications of genetic testing (Allen et al. 2016; Milo Rasouly et al. 2021; Ormond et al. 2019; Godino et al. 2021a). Misinterpretations of genetic risk may prevent individuals from making informed decisions about their health and from effectively communicating relevant genetic information to at-risk relatives. In this context, studying public perceptions of genetic communication and identifying potential misunderstandings is crucial to designing effective interventions that improve cascade testing uptake.

In order to explore these challenges in the general population and inform the future planning of initiatives aimed at improving the uptake of cascade testing in our country, we designed a questionnaire that investigates attitudes and preferences in the general Italian population on the communication of genetic risk information within families. Ensuring that the questionnaire was clear and accessible was a priority, as effective survey instruments must be designed to minimize misinterpretations and accurately capture public perspectives. Given the complexity of genetic concepts, the use of qualitative methods to assess respondents’ comprehension was crucial.

Cognitive interviews are a well-established qualitative technique for evaluating and refining survey instruments, particularly in complex domains such as health and genetics (Willis et al. 2005; Willis 2015). This method allows researchers to identify significant discrepancies between how study authors intend questions to be understood and how respondents actually interpret them. Such discrepancies are commonly observed in health-related surveys (Drennan 2003; Egger-Rainer 2019; Vreeman et al. 2014) and can compromise data validity. By systematically analyzing participants' thought processes while responding to survey questions, cognitive interviews help detect ambiguous wording, unclear terminology, and unintended interpretations (Boeije & Willis 2013; Terwee et al. 2018).

The iterative nature of cognitive interviewing plays a crucial role in survey refinement. Multiple rounds of interviews enable the progressive improvement of questionnaire items, ensuring they align with respondents’ understanding and reducing potential misinterpretations (Howlett et al. 2018; LaPietra et al. 2020). This approach is particularly valuable when exploring sensitive topics such as genetics, as cognitive interviews conducted in a familiar environment may facilitate more thoughtful and accurate responses (Crawford et al. 2017; Farmer et al. 2022). Given that genetic information often requires a nuanced understanding, misinterpretations can significantly affect the validity of survey data. By refining survey questions through cognitive interviews, researchers can improve accessibility, enhance data accuracy, and ensure that the questionnaire effectively captures participants’ perspectives on genetic communication.

Within this framework, our study aimed to refine our questionnaire through cognitive interviewing to improve its clarity and comprehensibility. By actively involving members of the general population, we sought to ensure that the instrument was appropriately structured, easily understandable, and effective in assessing public attitudes toward the communication of genetic risk within families.

## Methods

### Design

This was a qualitative study that involved cognitive interviews to assess the items of a questionnaire on the attitudes and preferences of the Italian general population on the communication of potential hereditary conditions to family members. The methodology was selected in light of its capacity to guide respondents through the questionnaire items asking them to reword the items, share their thoughts and feelings in reading them, and suggest additional terms to refer to the same concepts. This process aims to develop a set of questionnaire items that are clear, unambiguous and understandable for the target population.

### Participants

The study population consisted of members of the general population in Italy; a convenience sampling method (Golzar et al. 2022) was used for its practicality and efficiency in recruiting within a limited timeframe. Recruitment was conducted through informal networks and personal referrals, with participants being invited to take part in the study by individuals who were already aware of the research. This process occurred in two rounds: the first in October 2024 and the second in November 2024. Eligibility criteria were: being over 18 years of age, being an Italian citizen, having the ability to speak Italian and agreeing to participate. To ensure that responses reflected perspectives from the general population without prior exposure to genetics, individuals with a personal or family history of genetic conditions, as well as those working in the field of genetics, were excluded. Participants with diverse educational backgrounds were included to capture a broad range of perspectives and enhance the generalizability of the findings.

### Development of the questionnaire

The process involved several key steps. First, a systematic review was conducted to gain a comprehensive understanding of the topic and establish whether validated instruments were available in the literature. The quantitative studies identified (Godino et al. 2025) utilized a range of data collection instruments to explore attitudes toward genetic risk disclosure. However, all of these surveys were designed ad hoc, as shown in Table 1.

**Table 1:** Data collection instrument description of quantitative included studies in the Systematic Review (Godino et al. 2025)

Author, Date	Development of the Instrument	Instrument Features
Andersson et al. 2020	The questionnaire designed was first reviewed by experts in nursing, clinical genetics, ethics and oncology. Then it was tested in a brief pilot study (11 men and 14 women aged 21–74) The survey was made available from September to October 2018	Four different hypothetical scenarios were designed. Two of the scenarios described a familial cancer situation, where the lifetime risk of CRC was presented as being around 10% (moderate risk). The two other scenarios described a Lynch syndrome situation with hereditary CRC where the lifetime risk was presented to be around 70% (high risk). Each scenario was composed of six questions: five adjacent multiple-choice questions requiring one checkbox response per question, or in some items a text answer option and one open-ended comment box as a final item
Heaton & Chico 2016	An online survey was created, presenting a set of vignettes showing a range of possible disease scenarios The survey was made available from October to December 2013	The disease characteristics chosen to vary between vignettes were informed by preliminary focus groups (16 females and 8 males aged 20 to 70). The main themes identified to vary in each vignette were seriousness of the condition, the absolute risks of disease manifestation for the at-risk relative both before and after the test on the proband was performed, and the possibility of disease prevention. There were created 54 different scenarios
Maxwell et al. 2009	A pilot study of 50 telephone interviews tested aspects of instrument design. The final study was conducted using the same version of the survey instrument The survey was conducted in June 2008	Questions of the survey regarded: willingness to be informed of familial risk and be offered free testing, acceptability of different methods of contact, and reported likelihood of visiting a clinic for cascade screening
Petersen et al. 2019	Instrument development: NA Enrolled period: NA	The questionnaire contained five key questions. Each question was composed by three different choices (“yes”, “no” or “don’t know”, except for question two which showed different option for preference in disclosure)
Phillips et al. 2023	The survey was initially drafted in English and then translated in Dutch The survey was made available from July and August 2020	The questionnaire consisted of three main parts, namely vignettes, general questions from the perspective of the relative receiving genetic information, and questions regarding different international policy approaches to address nondisclosure of genetic risk. Two separate cases, colon cancer and Huntington’s disease, were described
Tiller et al. 2024	The survey was initially designed by a team of researchers. Before full launch, it was piloted on 100 respondents, and the data was reviewed to ensure consumer understanding Enrolled period: NA	After an introduction to the concept of medically actionable genetic conditions and the importance of sharing information, respondents were provided with two example letters, one with more specific information than the other The questionnaire was composed by either multiple choice questions or open-ended questions
Wolff et al. 2007	All questionnaires used were previously tested in pilot work Enrolled period: NA	Eight different disease scenarios were constructed, systematically varying three disease characteristics: fatality, penetrance, and availability of treatment. Knowledge about genetics was measured with seven statements where participants had to answer to be true or false, or whether they were uncertain. Knowledge about genetic testing was measured by having participants indicate on a five-point Likert-like scale how much they had heard about genetic testing for diseases. The scale was anchored by 1, ‘‘I have never heard of it,’’ and 5 ‘‘I am well informed about it
Marleen van den Heuvel et al. 2020	Instrument development: NA The survey was made available from February and April 2018	The surveys contained multiple-choice items, complemented by open answer items. Two vignettes were included: one with a treatable condition (ovarian cancer) and the other with an untreatable condition (Alzheimer’s disease). These were used to inform participants about hereditary diseases, inheritance patterns, and possible preventive or treatment and reproductive options

NA: Not available; CRC: colorectal cancer

^*^Godino et al. (2025)

Given the absence of a validated tool, an ad hoc questionnaire with an initial pool of items was developed, and the appropriate format for measurement was evaluated. To ensure content validity, an expert review of the initial item pool was conducted, involving two geneticists with expertise in cascade testing, a genetic nurse and a bioethicist with a specific interest in cascade testing; all had over 10 years of experience in their respective fields.

The questionnaire included multiple response formats tailored to the nature of the questions. Likert scale ranges, binary ‘yes/no’ options, trichotomous choices (yes/no/don’t know), and open-ended fields, offering a balanced approach for capturing both quantitative and qualitative data. The Likert range allows participants to convey varying levels of agreement or perception, which is particularly valuable in assessing sensitive attitudes toward genetic risk disclosure. On the other hand, binary and trichotomous response options provide clarity and reduce ambiguity for questions where a definitive choice or degree of certainty is essential. These formats were chosen to enhance clarity, reduce ambiguity, and facilitate precise categorical analysis, thereby minimizing interpretation bias (Dillman et al. 2016). Nuanced situations, such as instances in which relatives may prefer not to share genetic information, are addressed using open-ended items.

### Questionnaire

The questionnaire is structured into eight sections, each designed to explore specific aspects related to genetic conditions, testing, and communication, as detailed in Table 2. These sections include topics such as general knowledge of genetic testing and its purposes, personal and family experiences with hereditary diseases, and hypothetical scenarios regarding genetic predispositions. Additionally, the questionnaire investigates moral responsibilities and communication preferences, focusing on methods, mediators, and the context of delivering genetic risk information. It also addresses decisions related to the disclosure of personal genetic diagnoses and their impact on family relationships, assessed through the Italian version of SCORE-15 by Paolini and Schepisi (2020). Lastly, socio-demographic characteristics are collected to provide a comprehensive understanding of the participants’ background.

**Table 2:** Questionnaire: sections and explored concepts

Section	Explored Concepts
Genetic Conditions and Genetic Testing	General knowledge of genetic testing; Purposes of genetic testing (disease risk, treatment, drug efficacy, hereditary risk)
Personal and Family Experience	Presence of hereditary diseases in the family; Relationships with affected family members; Use of genetic testing within the family; Personal or family role in genetic testing
Hypothetical Scenarios of Hereditary Diseases	Preferences regarding knowledge of hypothetical genetic predisposition (Cystic Fibrosis, BRCA-related cancer risk, and early-onset Alzheimer’s disease); Reasons for wanting or not wanting to know; Decision-making for genetic testing
Moral Responsibility	Opinions on who should inform about genetic risks (family members, healthcare professionals, oneself); Roles and responsibilities in genetic risk communication
Communication Preferences	Preferred methods of communication (family vs. healthcare professional); Contact methods (in person, phone, email); Communication context (challenges, family relationships)
Disclosure of Personal Diagnosis	Decisions about sharing genetic information with family members; Preference for healthcare professionals' involvement in family communication
Family Relationships	The Italian version of SCORE-15 by Paolini & Schepisi 2020*
Socio-demographic Characteristics	Age, gender, marital status, education level, occupation, region of residence, religious approach

^*^Paolini D, Schepisi L. The Italian Version of SCORE-15: Validation and Potential Use. Fam Process. 2020 Dec;59(4):1789–1800. https://doi.org/10.1111/famp.12495

### Data collection procedure

All interviews were conducted remotely to allow participants to respond from a familiar environment, a setting known to enhance response quality by reducing anxiety and promoting reflection (Crawford et al. 2017; Farmer et al. 2022). On the day of the interview, participants were first asked to complete the questionnaire, after which they participated in a semi-structured interview with a genetic nurse who is a European Board of Medical Genetics registered genetic counsellor and has over 10 years of clinical experience. During the interview, each item was systematically evaluated to identify potential challenges, such as difficulty in responding, lack of clarity, or ambiguous wording. For each of these items, the genetic nurse evaluated items for potential challenges during the interview (e.g., whether they found the wording confusing, or difficult to understand or hard to answer), clarity, understandability, and potentially shocking or offensive aspects. Participants were then explicitly asked to suggest alternative phrasings for each item, in order to evaluate whether the developers' intended meaning was understood. They were also asked whether the instructions were understandable and whether the display and the length of the questionnaire were acceptable. At the end of the interview, additional comments about the questionnaire content were encouraged.

Then, data collection continued through two rounds of cognitive interviews, with saturation reached when no new themes, issues, or significant insights emerged from the participants' feedback in each round (Fusch and Ness 2015; Saunders et al. 2018). Saturation was defined as the point where the data provided by additional participants consistently confirmed previously identified patterns without introducing novel information or challenges (Saunders et al. 2018). After the first round of interviews, participant feedback was analysed, and the questionnaire was revised accordingly. The revision included a re-organization of the questionnaire and rewording of specific items to address previously identified problems, incorporating direct input from participants regarding how questions should be phrased to enhance clarity and accuracy. A new sample of participants from the general population was then recruited to assess the revised questionnaire. Through the results of the two rounds of interviews, we reached the final version of the questionnaire, following the methodological framework outlined by Howlett and colleagues (2018) and LaPietra and colleagues (2020). Supplementary Table S1 provides examples that illustrate the evolution of the questionnaire, as referenced in the Results section to support the described changes.

### Data analysis

The first step for the analysis and interpretation of the individual interviews was the transcription of the audio recordings. All the recordings were transcribed verbatim (by VG), with names and other identifying material altered to ensure confidentiality. Transcriptions were checked by LG, who also listened to the audio recordings. Conventional content analysis, as outlined by Hsieh and Shannon (2005), was applied to the interview transcripts to systematically identify, code, and categorize aspects of the data. This qualitative approach enabled a detailed exploration of participants’ understanding and perceptions of the questionnaire items, allowing for the emergence of themes directly grounded in the data. By forgoing predetermined categories, this inductive method ensures that the coding process remains aligned with the participants' actual expressions and experiences, thereby enhancing the reliability of insights related to the questionnaire items. The approach involved an initial thorough reading of the transcripts to establish familiarity with the data, followed by iterative coding to capture key problems experienced by the participants. Differences in opinions concerning the coding were solved through discussion among the authors. This iterative process continued until data saturation was reached, meaning that no new major comments by new participants interviewed emerged from the data.

### Ethical considerations

The study followed the principles of the Declaration of Helsinki and obtained ethics approval from the Ethics Committee of the University of Bologna on September 30th, 2024 (approval number 0313516 of 11th October, 2024). All of the participants were informed about the details of the study, including that participation was voluntary. They provided written informed consent, acknowledging their right to withdraw at any time. All collected data were handled confidentially and in compliance with privacy law.

## Results

### Participants

Two rounds of cognitive interviews were conducted to refine the questionnaire. In the first round, data saturation was achieved after 30 participants, while in the second round, saturation was reached with 20 participants.

In the first round, participants (n = 30) had a mean age of 37.3 years (range 18–82), and 73.3% were women. In terms of educational level, 3.3% had completed elementary school, 6.7% had a middle school diploma, 43.3% a high school diploma, 16.7% a university degree, and 30.0% a post-graduate specialization. As for employment status, 63.3% reported paid employment (Table 3).

**Table 3:** Characteristics of participants

	Round 1 (N = 30)		Round 2 (N = 20)	
	n	%	n	%
Gender				
*Male*	8	26.7	8	40.0
*Female*	21	70.0	12	60.0
*Prefer not to say*	1	3.3	0	0
Education level				
*Elementary school*	1	3.3	0	0
*Middle school diploma*	2	6.7	2	10.0
*High school diploma*	11	43.3	3	15.0
*University degree*	10	16.7	8	40.0
*Post-graduate specialization*	6	30.0	7	35.0
Marital status				
*Single*	18	60.0	8	40.0
*Married*	5	16.7	7	35.0
*Cohabiting*	5	16.7	4	20.0
*Divorced/Separeted*	1	3.3	1	5.0
*Widowed*	1	3.3	0	0
Having children				
*Yes*	8	26.7	9	45.0
*No*	22	73.3	11	55.0
Region of residence				
*Emilia-Romagna*	15	50.0	14	70.0
*Lazio*	9	30.0	5	25.0
*Marche*	2	6.7	0	0
*Puglia*	1	3.3	0	0
*Sicily*	2	6.7	1	5.0
*Umbria*	1	3.3	0	0

In the second round, participants (n = 20) had a mean age of 38.2 years (range 18–67), and 60.0% were women. Regarding educational level, 10.0% had a middle school diploma, 15.0% a high school diploma, 40.0% a university degree, and 35.0% a post-graduate specialization. Concerning employment status, 70.0% were employed (Table 3).

### General problems identified in the questionnaire

All of the major problems were identified during the first round of cognitive interviews and had to do with the questionnaire instructions, questions wording, and the sensitive nature of the topics. After implementing revisions based on this initial feedback, the second round of interviews confirmed that these changes had improved clarity and reduced ambiguity, as only minor issues emerged (e.g., inconsistencies in gendered language and minor typographical errors). These issues were resolved without substantially changing the questionnaire. Overall, the feedback provided by the participants in the second round indicated that the revised version was easier to understand while at the same time capturing what we intended.

Qualitative analysis of the first round identified four key themes reflecting participants’ challenges in understanding the questionnaire: difficulties with genetic terminology, ambiguities surrounding genetic testing and the concept of family, confusion between genetic risk and disease diagnosis, and the conflation of authorization and responsibility in genetic communication.

### Challenges in understanding genetic terminology

The analysis revealed significant challenges in participants’ understanding of key genetic terminology, particularly gene names, which were frequently perceived as complex scientific jargon. Participants often described these terms as 't*echnical*' or '*scientific*,' reporting feelings of anxiety or fear when encountering them. This reaction highlights the sensitivity, as participants associated such terminology with serious health conditions, which heightened their emotional response and created additional barriers to comprehension. Specifically, participants stated:

*“I find the terms too scientific and would prefer simpler ones”*

*id20, Female, 82 years, elementary school*

*“Unexplained acronyms, clinical cases involving less commonly known conditions, and the need to reread scenarios multiple times due to my lack of expertise in the field”*

*id29, Male,31 years, post-graduate specialization*

*“Removing or explaining the meaning of scientific terms would make the text easier to understand”*

*id15, Female, 22 years, middle school diploma*

*"At first glance, seeing the name of the gene can set off an alarm bell, and the initial thought might be, 'Oh no, what am I reading? What is this?'"*

*id27, Female, 42 years, post-graduate specialization*

This response suggests that gene names and scientific terms may interfere with understanding. To address this, the technical nature of these terms was reduced, and accessible explanations were provided in public-facing genetic information to mitigate apprehensive responses and promote a more accessible understanding of genetic concepts. Examples of these adjustments to the questionnaire are presented in Supplementary File Table S1.

### Ambiguities surrounding the concepts of ‘genetic testing’ and ‘family

A second major theme was the ambiguities regarding the terminology used within the questionnaire, particularly around the concept of 'genetic testing.' In some cases, participants interpreted genetic testing as a consultation or interview rather than DNA analysis performed by a laboratory. Specifically, participants stated:

*“Genetic test: I’m not sure what it involves (whether it’s like a blood test or an interview), but I understand what it’s for. The words themselves are clear, but I don’t have much information about it.”*

*id15, Female, 22 years, middle school diploma*

*“I would suggest including a brief introduction using very simple language to explain what you mean by genetic test. This would provide participants with the tools they need to be able to answer the questionnaire.”*

*id31, Female,59 years, post-graduate specialization*

Additionally, confusion arose over the term 'family'. Participants did not understand whether it referred specifically to bloodline relatives, the immediate family, or included extended family members. Some even wondered whether the term 'family' could include the partner's family. Specifically, a young woman stated:

*"You should define what you mean by family: immediate family or extended family? It would also be helpful to include some examples. Does it refer to my family or to my partner’s family too?"*

*id07, Female, 23 years, university degree*

In one case, a female participant wondered whether she should consider her daughter as a member of her ‘family’, based on a genetic condition that the child inherited from her father, the participant’s husband. This inquiry reflects a sophisticated understanding of genetic risk.

*"I answered 'yes' to the question, 'Are you aware of any hereditary diseases in your family?' because my daughter is at risk of a hereditary condition, but it doesn’t come from my family … it’s from her father’s side. Now, talking this through with you, I’m wondering if I understood the question correctly. I thought of my daughter as part of my immediate family, which is why I answered 'yes.' To me, my daughter is my family!"*

*id13, Female, 54 years, high school diploma*

This observation highlights the complexity of defining ‘family’ in the context of hereditary conditions. For both these concepts, examples of adjustments to the questionnaire are presented in Supplementary File Table S1.

### Interpreting ‘genetic risk’ as a diagnosis

A third major theme identified was the frequent confusion regarding ‘genetic predisposition’ and ‘genetic risk.’ Many participants understood ‘genetic risk’ as an indicator of an existing disease rather than the likelihood of developing a disease associated with a familial PV. Specifically, participants stated:

*"Is a predisposition considered a hereditary disease? In my family, there is a predisposition to cancer."*

*id17, Female, 21 years, high school diploma*

*"The question 'Why wouldn’t you want to be informed about the presence of a variant in your family?' could be made clearer by replacing the term 'genetic variant' with 'disease'."*

*id08, Male, 26 years, post-graduate specialization*

The same question was paraphrased as:

*"If I want to be informed about the disease."*

*id10; Female,22 years, diploma; id11, Male, 59 years, high school diploma*

*"Why do you want to be informed about the disease I have?"*

*id 18, Male,33 years, high school diploma*

This misinterpretation led several respondents to equate genetic risk with an immediate health diagnosis, suggesting they interpreted the question as an inquiry about a relative’s active disease status, rather than the future implications of a familial PV. This conflation points to a substantial gap in understanding probabilistic concepts in genetics, highlighting the importance of clearer communication strategies that differentiate between disease and predisposition. Such clarity is crucial for ensuring that individuals accurately interpret genetic information and understand the implications in terms of potential future health risks rather than current pathology. Examples of these adjustments to the questionnaire are presented in Supplementary File Table S1.

### Ambiguities between ‘authorization’ and ‘responsibility’ in genetic communication

The analysis also uncovered considerable ambiguity surrounding the concepts of ‘authorization’ and ‘responsibility’ in the communication of genetic data. Participants frequently confused or used these terms interchangeably, revealing a lack of clarity in the procedural and ethical distinctions inherent in sharing genetic information. For example:

*"I don’t understand the term 'authorization' or the term 'responsibility' clearly."*

*id09, Female, 24 years, high school diploma*

*With regard to the specific question on responsibility, participants stated:*

*"The text itself isn’t difficult to understand; the challenge lies in the legal and bureaucratic aspects because I’m not sure who holds the legal responsibility."*

*id29, Male,31 years, post-graduate specialization*

*"For the question 'Who, in your opinion, is responsible for informing you about the diagnosis of a genetic condition in the family?' I would specify the term 'legal' to clarify that it refers to legal responsibility."*

*id32, Female,27 years, university degree*

With regard to the question on authorization, participants stated:

*“The question "Who, in your opinion, is authorized to inform you about the diagnosis of a genetic condition in the family?" can be rephrased as "Who is allowed to inform me about the diagnosis?"”*

*id16, Female, 50 years, post-graduate specialization*

There was confusion regarding who has the authority to disclose genetic information within a family and who holds the responsibility for doing so.

Additionally, a recurrent theme of uncertainty emerged around the terms "*privacy*" and "*confidentiality*". Several participants expressed difficulty distinguishing between these concepts, further complicating their understanding of ethical boundaries in genetic information sharing:

*"I don’t understand the difference between privacy and confidentiality."*

*id27, Female, 42 years, post-graduate specialization.*

*"I don’t know the difference between privacy and the principle of confidentiality."*

*id15, Female, 22 years, middle school diploma.*

These findings highlighted the need for clear, accessible definitions to clarify not just the roles of authorization and responsibility but also the difference between privacy and confidentiality. These concepts are crucial to enable the sharing of genetic information within families in accurate and ethically responsible ways. Considering the aforementioned challenges, the authors critically reflected on the relationship between responsibility and authorization. They concluded that while the concept of responsibility (moral responsibility) is both relevant and something people are able to understand, the notion of legal authorization is less relevant and not something that the general population might be necessarily familiar with. For these reasons, the decision was made to remove references to legal authorization and retain the emphasis on responsibility. Similarly, the concepts of privacy and confidentiality were rephrased to enhance clarity and align more closely with the participants' comprehension, ensuring that the questionnaire employed terminology that was both accurate and accessible. Examples of these adjustments to the questionnaire are presented in Supplementary File Table S1.

## Discussion

This study aimed to improve the comprehensibility and clarity of a questionnaire on the attitudes and preferences of the Italian general population on the communication of genetic risk information within families.

In order to reach our goal we employed cognitive interviews, a valuable approach used in the fields of health, education, and social sciences as an analytical tool aimed at identifying misinterpretations of survey items (Boeije and Willis 2013; Terwee et al. 2018; Willis et al. 2005; Willis 2015). The iterative nature of cognitive interviews plays a pivotal role in improving survey instruments. Research by Howlett and colleagues (2018) and LaPietra and colleagues (2020) demonstrated that conducting multiple rounds of interviews facilitates the systematic improvement of survey items, ensuring they align more effectively with respondents’ understanding. Additionally, field-based cognitive interviews also add to data validity (Crawford et al. 2017; Farmer et al. 2022). This is especially important in surveys with the general population when dealing with complex and sensitive topics such as genetics, which require nuanced understanding.

With the active involvement of the lay public, we were able to refine question wording, restructure ambiguous items, and ensure that key concepts were clearly conveyed. The most significant revisions were made after the first round of interviews, where participants identified challenges related to genetic terminology, misinterpretations of genetic risk, and confusion about the scope of family in a genetic context. By addressing these issues, the revised questionnaire became more accessible and aligned with the general population’s understanding, reducing the likelihood of misinterpretations affecting survey responses. The conceptualization of genetic risk as an actual disease may lead to important misinterpretations of the utility of genetic testing and/or preventive measures, highlighting the importance of refining the questionnaire to ensure that key concepts are clearly differentiated. The iterative process of testing performed was found to be highly effective, as demonstrated by the fact that no comprehension difficulties were reported in the second round of interviews.

Our results revealed that the problems identified could be attributed to low levels of genetic literacy and a complex and varied understanding of the concept of “family” in the context of genetic risk communication. Regarding genetic literacy, the difficulties encountered with gene names and scientific terms, often perceived as unsettling and worrisome, may hinder comprehension, and thus interfere with the sharing of genetic information within the family. The misconception that genetic risk equates to a definitive diagnosis may further complicate discussions within families, potentially leading to either unnecessary alarm or the dismissal of genetic risk as irrelevant.

Beyond issues of genetic literacy, our findings also highlight how family is conceptualized differently by healthcare professionals and the general public, influencing the communication of genetic risk. While healthcare professionals and researchers often define family in biological terms, study participants demonstrated a more fluid and socially constructed understanding of family relationships. For example, some questioned whether a partner’s relatives should be considered "family" in the context of sharing genetic risk information. These findings align with previous research on the distinction between biological and social definitions of family (Edwards et al. 2012; Edwards and Gillies 2012) and suggest that a rigid biomedical approach to family structures may not fully capture how individuals navigate intrafamilial communication of genetic risk.

The way individuals define family also shapes their perceptions of responsibility in communicating genetic information. If someone does not consider certain relatives as part of their immediate family, they may feel less obligated to inform them of potential hereditary risks. This emphasizes the need for healthcare professionals to acknowledge and address diverse family structures when providing genetic counseling and risk communication guidance.

At the same time, these results underscore the necessity for targeted educational interventions that can bridge the knowledge gap and enhance public understanding of genetic concepts. As highlighted by Carere and colleagues (2016), improving genetic literacy is essential for empowering individuals to make informed health decisions. However, it is important to consider that the extent to which our findings on genetic literacy apply to the broader population is limited by the characteristics of our sample. Individuals with lower levels of education or health literacy may face even greater difficulties in understanding genetic concepts, which could further influence their ability to communicate genetic risk within the family. Future research should explore tailored educational strategies that address specific misconceptions and improve public engagement with genetic information.

Our findings also revealed misconceptions regarding the concepts of "responsibility" and "authorization" in the context of familial communication of genetic risk. If responsibility of genetic risk communication is perceived as a legal responsibility (and not a moral obligation), the person who holds the genetic information may be more reluctant to take the burden of engaging a conversation about genetics with family members. These misunderstandings may result in inadequate awareness and comprehension of genetic risks among family members. Evidence from Metcalfe and colleagues (2011), Godino and colleagues (2018; 2019; 2021b), and Di Pietro and colleagues (2021) underscores the critical role of family communication dynamics in shaping the dissemination of genetic risk information. By acknowledging the diversity in how individuals perceive family and responsibility, interventions can be designed to support more effective and inclusive risk communication strategies.

These considerations highlight the importance of employing suitable methods to ensure that survey instruments exploring genetic issues accurately capture the perspectives of the public population. In our study, the significant reduction in the number of problems identified by new participants in the updated version of the questionnaire showed that cognitive interviewing proved to be useful for improving the questionnaire in many different ways. In line with other experiences with cognitive interviews used in the general population (Fuller et al. 2017; Malpass et al. 2016; Watt et al. 2008).

This study has some strengths. To the best of our knowledge, it is the first qualitative study involving cognitive interviews to refine a questionnaire that investigates the attitudes and preferences of the general population regarding the communication of potential hereditary conditions within families, with a focus on increasing clarity and comprehensibility. We employed rigorous qualitative research methods (Hsieh and Shannon 2005; Willis et al. 2005; Willis 2015) and adhered to established standards for qualitative research (Tong et al. 2007), using techniques to ensure the study’s rigor (Barbour 2001). The data were transcribed *verbatim* and meticulously verified through double-checking of the audio recordings. Independent coding by two authors ensured that all data were given equal consideration and reanalysis of the original dataset after coding allowed for thorough collation of all coded items and careful review of themes. Furthermore, the research team included experts from diverse backgrounds, fostering a multidisciplinary approach to data interpretation and analysis. Moreover, conducting multiple rounds of cognitive interviews played a pivotal role in facilitating the systematic improvement of our questionnaire items (Howlett et al. 2018; LaPietra et al. 2020). However, several limitations need to be acknowledged. Although convenience sampling is a frequently used method for acquiring participants in qualitative research, selection bias may have affected the findings. While we included participants with diverse educational backgrounds, the sample may not fully reflect the characteristics of the general Italian population. In particular, individuals with lower educational levels, who may struggle more with understanding genetic concepts, may have been underrepresented. This limitation is particularly relevant given that one of the main themes identified in our study was genetic literacy. Future research should aim to include a more representative sample, possibly through stratified sampling methods, to better assess how different sociodemographic factors influence genetic literacy and communication within families. However, individuals who accept to participate in this type of research may be especially interested in research and more careful with their answers than the actual survey respondents as a whole, which may not always reflect real life.

## Conclusion

Our findings highlight the importance of assessing concept clarity and comprehension in questionnaire development for the general population, especially in the case of sensitive areas such as genetic health. While many survey studies report piloting their questionnaires before distribution, this study represents a rigorous and systematic effort to refine a survey instrument through multiple iterations of cognitive interviewing, actively engaging the lay public to ensure that the questions were interpreted as intended.

Study participants perceived technical names as a barrier, and common genetic terms such as genetic testing and genetic risk were construed in different ways. This highlights a broader issue in survey research: are we truly measuring what we intend to measure? The iterative approach used in this study not only improved the clarity, accessibility, and usability of the questionnaire, but also resulted in an instrument that was more tailored to the needs of the target population.

Beyond the development of a more effective questionnaire, our findings contribute to a growing body of research emphasizing how expert conceptualizations and lexicon often diverge from public understanding, an issue that other researchers in health communication and survey methodology should consider. Addressing this gap is particularly relevant when studying genetic literacy and risk communication, as misunderstandings in these areas can significantly impact decision-making within families.

In conclusion, we believe that the developed questionnaire represents a good instrument for collecting reliable and meaningful data from the Italian general population. We hope that our work encourages further research on the alignment between expert and public interpretations of genetic information and that this instrument will facilitate future studies, contributing to improved understanding and more informed decision-making about potential hereditary conditions.

## Supplementary Information

Below is the link to the electronic supplementary material.

Supplementary file1 (DOCX 20 KB)

## Data Availability

The data that support the findings of this study are available from the corresponding author upon reasonable request.
