# Supplementary material for: Developing a questionnaire to explore lay people’s preferences for communicating hereditary conditions within families: insights from a cognitive interview study
Source: J Community Genet. 2025 Mar 18;16(3):351–62. doi: 10.1007/s12687-025-00783-6 (PMC12202257; doi:10.1007/s12687-025-00783-6)
Supplement: Supplementary file 1 — Supplementary file1 (DOCX 20 KB) [file 12687_2025_783_MOESM1_ESM.docx]

**Supplementary File**

**Table S1**. Examples of text adaptation based on cognitive interviews (Willis et al., 2005; Willis, 2015)

| **Major themes** | **Original text** | **Adapted final text** |
| --- | --- | --- |
| Challenges in Understanding Genetic Terminology (question component) | Imagine that one of the following situations occurs in your family:  *Scenario 1*  After having a child affected by Cystic fibrosis, an uncle of yours, who lives in another city, discovers that he is a healthy carrier of a variant in the CFTR gene.  *Cystic Fibrosis is a genetic disease that primarily affects the lungs, and causes recurrent respiratory infections, as well as the digestive system. Healthy carriers of Cystic Fibrosis generally have no symptoms, but if they have children with another carrier, the children may be affected by the disease.*  *Scenario 2*  An aunt of yours, who lives in another city, is diagnosed with ovarian cancer, and tests reveal that she carries a variant in the BRCA2 gene.  *People with inherited alterations in the BRCA genes have an increased risk of developing breast and ovarian cancer (in women), prostate cancer (in men), and other types of cancer. For this reason, they undergo appropriate surveillance and preventive measures for high-risk individuals.*  *Scenario 3*  An uncle of yours, who lives in another city, develops Alzheimer's disease, and tests reveal that he carries a variant in the PSEN1 gene.  *Carriers of genetic variants in the PSEN1 gene develop an early-onset form of Alzheimer's that is currently not preventable. This is a very rare condition: the vast majority of Alzheimer's cases is* ***NOT*** *hereditary.* | Imagine that one of the following situations occurs in your family:  *Scenario 1*  A cousin of yours is diagnosed with Cystic Fibrosis. Your aunt and uncle then discover that they both carry a genetic variant that causes the disease.  *Cystic Fibrosis is a serious genetic disorder that primarily affects the lungs and the digestive system. Parents who carry one copy of a disease-causing genetic variant have no symptoms, but they can pass it on to their children. If both parents are carriers, their children may inherit the disease. Knowing their carrier status enables couples to make informed decisions about family planning. In Italy, about 1 in 25 people is a healthy carrier of a genetic variant that causes Cystic Fibrosis.*  *Scenario 2*  One of your aunts has ovarian cancer and has been found to carry a genetic variant that increases the risk of developing cancers such as breast, ovarian and prostate cancer.  *People with this genetic variant are at high risk and can benefit from surveillance and prevention programs to facilitate early diagnosis and reduce the likelihood of developing cancer. In Italy, approximately 1 in 500 people has a genetic variant that increases the risk of these types of cancer.*  *Scenario 3*  An uncle of yours shows signs of Alzheimer's. Tests reveal that he carries a genetic variant that causes an early-onset form of the disease.  *Almost all carriers of this type of genetic variant develop the disease, and no preventive measures or medications are currently available that can lower their risk. This is a very rare condition: the vast majority of Alzheimer's cases are* ***NOT*** *directly hereditary.* |
| Ambiguities Surrounding the Concepts of ‘Genetic Testing’ and ‘Family’  (Instruction component) | In these three scenarios, you have a 25% chance (1 in 4) of having the same genetic variant found in your aunt or uncle. | Imagine that in these three scenarios, your aunt and uncle are your mother’s siblings, so you have a 25% chance (1 in 4) of having the genetic variant that runs on your mother’s side of the family. |
| Misinterpretations of ‘Genetic Risk’ as Disease Diagnosis (Instruction component) | *Thank you for answering the questions about three hypothetical scenarios. Now, moving on, we ask you to answer the following questions about who you think can or should inform you of a genetic diagnosis in the family.* | *Thank you for answering the questions about the three scenarios. Now, we ask you to respond to some questions about* ***who is morally responsible*** *of informing you about the genetic risk that runs in your family. Consider that when a genetic variant is found in a person with a disease, doctors usually recommend that the person share that information with their relatives so they, in turn, can seek specialist advice to better understand their own risk. Sharing this information with family members can be complicated or difficult. The result is that relatives are often not informed.* |
| Ambiguities Between ‘Authorization’ and ‘Responsibility’ in Genetic Communication (question component) | 4. Who do you think has the responsibility to inform you?  4a. Family members who know about the genetic diagnosis Yes/No/Not sure  4b. Doctors/healthcare providers who made the genetic diagnosis Yes/No/Not sure  5. Who do you think is authorized to inform you?  5a. Family members who are aware of the genetic diagnosis Yes/No/Not sure  5b. Doctors/healthcare providers who made the genetic diagnosis Yes/No/Not sure | 4. In your view, who has the moral responsibility to inform you?  4a. I am morally responsible for seeking the information myself Yes/No/Not sure  4b. My family members Yes/No/Not sure  4c. The doctors/healthcare professionals Yes/No/Not sure  4d. Others (please specify) ……………………………… |
